# Distribution of the Quill Mite *Bubophilus aluconis* Parasitising Western Palaearctic Owls Belonging to the Genus *Strix*

**DOI:** 10.1155/2024/6110049

**Published:** 2024-10-08

**Authors:** Zbigniew Kwieciński, Jakub Z. Kosicki, Maciej Skoracki

**Affiliations:** ^1^Department of Avian Biology and Ecology, Faculty of Biology, Adam Mickiewicz University, Uniwersytetu Poznańskiego 6 61-614, Poznań, Poland; ^2^Department of Animal Morphology, Faculty of Biology, Adam Mickiewicz University, Uniwersytetu Poznańskiego 6 61–614, Poznan, Poland

**Keywords:** Acari, ectoparasites, great grey owl, host-parasite relationship, quill mites, Syringophilidae, tawny owl, Ural owl

## Abstract

**Background:** The paper presents the results of studies on the distribution of the parasitic quill mite species *Bubophilus aluconis* (Acariformes: Syringophilidae) in the feather quills of the Western Palaearctic owls of the genus *Strix*, that is, tawny owl *S. aluco*, collected in Poland and Sweden, the Ural owl *S. uralensis*, from Poland, Sweden, and Finland, and the great grey owl *S. nebulosa*, from Sweden and Finland. The two latter species are new hosts for *B. aluconis*.

**Methods:** Feather samples of the tawny owl, Ural owl, and great grey owl used in this study come from Prof. Marian Cieślak's private collection, which contains feathers of Western Palearctic birds of prey and owls. Each of the 77 tawny owl, 75 Ural owl, and 55 great grey owl specimens are represented either by whole dry wings or a full complement of flight feathers (primaries (P) and secondaries (S)) and tails (rectrices (R)). Particular types of feathers were coded as follows: Ps, Ss, R, primary greater upperwing coverts (PGUppC), secondary greater upperwing coverts (SGUppC), primary greater underwing coverts (PGUndrC), secondary greater underwing coverts (SGUndrC), uppertail coverts (UppTC), and undertail coverts (UndrTC).

**Results:** The prevalence was relatively low for all of the examined birds, that is, 12% for *S. uralensis* (*N* = 79), 2.6% for *S. aluco* (*N* = 77), and 3.6% for *S. nebulosa* (*N* = 55). In total, we examined 37,260 flight feathers and coverts. The mite *B. aluconis* occupied only the inner S of the tawny owl, secondary greater under and upperwing coverts of the great grey owl, and inner Ss, primary and secondary upperwing coverts, UndrTC, and coverts from the scapulars of the Ural owl.

**Conclusion:** We hypothesise that the absence of these parasites in the examined P, S, and R suggests that these feather types might be unsuitable for the mites due to their thick quill walls, preventing successful feeding.

## 1. Introduction

Syringophilid mites (Acariformes, Prostigmata, and Syringophilidae) are a group of obligatory and permanent parasites living and reproducing within quills [1, 2]. The species of the family are highly host-specific, being mostly mono or oligoxenous parasites. They are also highly habitat-specific, inhabiting only a specific type of feathers [3, 4]. All stages of these mites feed on the fluids of the soft tissue of their hosts by piercing the quill wall with their needle-like chelicerae forming a cheliceral tube [5]. Fertilised females, which are the dispersal stage, invade newly forming feathers, where they start to produce eggs one at a time [2]. The dispersion is strictly correlated with the biology and ecology of hosts and syringophilids in three ways. First, during the host breeding season, the mites transmit vertically from infested feathers of parent birds to nestlings' juvenile plumage (nestling passage). Second, they can infest newly developed feathers on the same host during the fall moult (moulting passage). Third, according to indirect data, they can transfer horizontally between a “normal” host and its avian predator, from one adult to a conspecific adult, particularly during gregarious birds' moult and between sexes during copulation [6–11]. So far, about 400 species of Syringophilidae have been described, and the range of their avian hosts comprises 24 orders from all zoogeographical regions except Antarctica [12]. However, the biodiversity of syringophilid mites associated with owls is still poorly recognised, and they are known to include only seven species grouped into three genera, that is, *Bubophilus* (4 species), *Neobubophilus* (2), and *Megasyringophilus* (1) collected from 11 owl species both from the family Strigidae (10 species) and Tytonidae (1) [13–19].

In this paper, we present the results of our studies on the distribution of quill mites on Western Palearctic owls of the genus *Strix*: tawny owl *Strix aluco* Linnaeus, Ural owl *Strix uralensis* Pallas, and great grey owl *Strix nebulosa* Forster.

The tawny owl, the most common owl species in Europe, belong to the Palearctic faunal type, whose western group constitutes the European distribution element, while the eastern one is the Chinese-Himalayan element. This resident species occurs across Europe, except Iceland and the northern regions of Russia and Scandinavia. Western Europe is home to birds of the subspecies *S.a. sylvatica*, while *S.a. aluco* occur in the rest of the continent. The Ural owls, which belong to the Siberian faunal type, have a trans-Palaearctic distribution. This species inhabits northern and eastern parts of the continent and mountainous regions of Central and southeast Europe. The contiguous main range in northeast Europe is occupied by *S.u. liturata*, while *S.u. macroura* occurs in the southeast. The great grey owl is the least abundant species of the genus *Strix*, and its distribution is dispersed—even though it can be locally dense—in the boreal part of the Western Palearctic. Like the Ural owl, the great grey owl belongs to the Siberian faunal type but occupies a vast circumpolar range in boreal climatic zones and boreal mountainous regions. Incidentally, this is the only species of *Strix* that occurs both in Eurasia and North America. Great grey owls breed in north-eastern Europe, approaching the range of the Snowy Owl *Bubo scandiacus* in the north [20–23].

Based on the biology and behaviour of the owl of the genus *Strix*, below we present the potential routes of quill mite infestation and, thus, the possibility of transfer between hosts. We discuss the possibilities of mite distribution and dispersion in plumage and habitat preferences of quill mites.

## 2. Materials and Methods

Feather samples of the tawny owl, Ural owl, and great grey owl used in this study come from Prof. Marian Cieślak's private collection, which contains feathers of Western Palearctic birds of prey and owls [24]. Currently, it is deposited in the Department of Avian Biology and Ecology, Faculty of Biology, Adam Mickiewicz University, Poznań, Poland (AMU-DABE). Individuals of a given species have been collected over many years in many geographical locations in Europe. Details on the collection and methods of collecting feathers, origin, and identification of sex and age of the studied owl species are contained in several publications [24–26]. Each of the 77 tawny owl, 75 Ural owl, and 55 great grey owl specimens are represented either by whole dry wings or a full complement of flight feathers (primaries (P) and secondaries (S)) and tails (rectrices (R)).

Particular types of feathers were coded as follows: P, S, R, primary greater upperwing coverts (PGUppC), secondary greater upperwing coverts (SGUppC), primary greater underwing coverts (PGUndrC), secondary greater underwing coverts (SGUndrC), uppertail coverts (UppTC), undertail coverts (UndrTC).

Mites were collected using a protocol proposed by Skoracki [3]. Each quill feather was examined under a dissecting microscope for evidence of quill mites. Infested feathers were opened, and mites were extracted using sharp, fine tweezers via a longitudinal cut in the quill made with a scalpel. Before mounting, mites were softened and cleared in Nesbitt's solution at 50°C for 1 h [3]. Then, mites were mounted on slides in Faure's medium [27]. Identifications were carried out with the ZEISS Axioskop2 light microscope (Carl-Zeiss AG, Germany), equipped with DIC optics. All collected mite specimens are deposited in the Adam Mickiewicz University, Department of Animal Morphology, Poznan, Poland (AMU). The prevalence and exact confidence limits for the prevalence (Sterne's method; confidence level = 95%) were computed using Quantitative Parasitology on the Web [28, 29].

## 3. Results

In total, 37,264 feathers from 207 individuals of the three species (tawny owl, Ural owl, and great grey owl) were examined. Some individuals of each owl species were infested with the quill mite species *Bubophilus aluconis* [14].

### 3.1. Prevalence and Feather Infestation of the Tawny Owl

A total of 77 (40 males, 37 females) specimens were examined, including birds from Poland, Germany, Czechia, Slovakia, Austria, Denmark, Sweden, and Finland. In total, 13,860 feathers were examined (Table 1). Among them, only two host specimens were infested by the quill mites making the index of prevalence, IP = 2.6%, and the confidence interval for prevalence (Sterne's method) (0.5–8.9) (Table 2).

**Figure 1:**
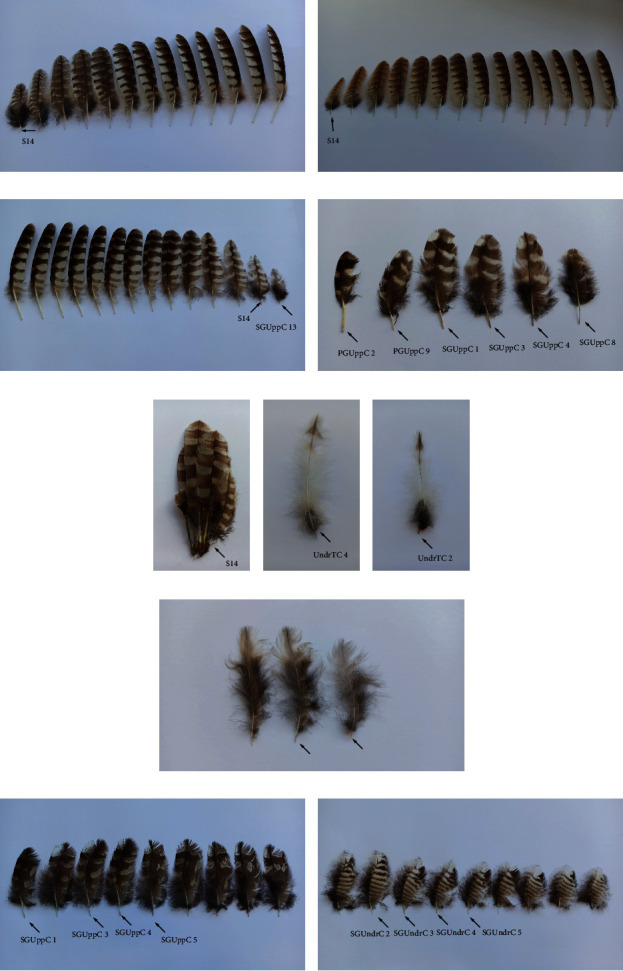
Feathers (marked by an arrow) infested with quill mites, *Bubophilus aluconis* sp.n. (a) The tawny owl (ad. female; host no. SA 7 from Poland), secondary - S 14 (grey morph). (b) The tawny owl (juv. Male; host no. SA 57 from Sweden), secondary - S 14 (brown morph). (c) The Ural owl (ad. female; host no. SUr 34 from Sweden), secondary - S 14 and secondary greater upperwing coverts: SGUppC no. 13. (d) The Ural owl (ad. female; host no. SUr 32 from Sweden), primary greater upperwing coverts: PGUppC no. 2, 9, respectively, and secondary greater upperwing coverts: SGUppC no. 1, 3, 4, 8. (e) The Ural owl (ad. male; host no. SUr 52 from Sweden), a fragment of the wing with secondaries - S 14. (f) The Ural owl (ad. male; host no. SUr 48 from Poland), undertail coverts: UndrTC no. 4 (right side). (g) The Ural owl (ad. female; host no. SUr 41 from Finland), undertail coverts: UndrTC no. 2 (left side). (h) The Ural owl (juv. female; host no. SUr 73 from Finland), two coverts from scapulars. (i) The great grey owl (ad. female; host no. SN 24 from Sweden), secondary greater upperwing coverts: SGUppC nos. 1, 3, 4, 5, respectively. (j) The great grey owl (ad. female; host no. SN 24 from Sweden), secondary greater underwing coverts: SGUndrC nos. 2, 3, 4, and 5, respectively.

In the material consisting of 37 juveniles (in the 1st calendar year (CY)) and 40 adults (in the 2nd CY or older), only one juvenile (IP = 1.3%) and one adult (IP = 1.3%) were infested by quill mites—an adult female from Poland with a grey morph and a juvenile male from Sweden with a brown morph. Quill mites were found in the single feather, inner S (S14), of two bird individuals (Figures 1(a) and 1(b)).

### 3.2. Mite Material Examined Collected From the Tawny Owl

Five females and two males are from the quill of small inner secondaries (host no. AMU-DABE SA 7, adult female); Poland: Kampinos, August 20, 2002, coll. M. Cieślak (Figure 1(a)).

Seven females, one male, and specimens were preserved in alcohol from the quill of small inner secondaries (host no. SA 57, juvenile male); Sweden: Kolmarden, August 26, 2005, coll. M. Cieslak (Figure 1(b)).

### 3.3. Prevalence and Feather Infestation of the Ural Owl

A total of 75 (38 males, 37 females) specimens were examined, including birds originating from Poland, Sweden, and Finland. In total, 13,500 feathers were examined (Table 1). A total of nine host specimens were infested by the quill mites, IP = 12%, 6.2–21.2 (Table 2).

In the material consisting of 36 juvenile (in the 1st CY) and 39 adult (in the 2nd CY or older) birds, only two juvenile (IP = 2.7%) and seven adult hosts (IP = 9.3%) were infested by quill mites, that is, a juvenile male from Finland, an adult female from Sweden, an adult female from Sweden, an adult female from Finland, an adult male from Sweden, an adult male from Poland, an adult male from Sweden, an adult male from Finland, and a juvenile female from Finland.

Quill mites were found in 17 individual feathers: in three small inner Ss: S 14 (host no. SUr 34, SUr 47, SUr 52), two in PGUppC no. 2, 9 (host no. SUr 32), eight in SGUppC no. 1, 3, 4, 8 (host no. SUr 32), SGUppC no. 3 (1-left, 1-right wings, no. SUr 66) and two no. 5 (host no. SUr 66, no. SUr 14), two - UndrTC no. 2 left side (host no. SUr 41) and UndrTC no. 4 right side (SUr 48), and two coverts from scapulars (host no. SUr 73) (Figures 1(c), 1(d), 1(e), 1(f), 1(g), and 1(h)).

### 3.4. Mite Material Examined Collected From the Ural Owl

Five females, one male, and specimens were preserved in alcohol from the quill of SGUppCs (host reg. no. SUr 14, juvenile male); Finland: Lahti, June 10, 2003, coll. M. Cieślak.

Three females and specimens were preserved in alcohol from quills of PGUppCs and SGUppCs (host no. SUr 32, adult female); Sweden: Vittjärv, September 23, 2005, coll. M. Cieślak (Figure 1(d)).

Three females and specimens were preserved in alcohol from quills of secondaries and SGUppCs (host no. SUr 34, adult female); Sweden: Vittjärv, August 14, 2005, coll. M. Cieślak (Figure 1(c)).

Two females and specimens were preserved in alcohol from the quill of UndrTC (host no. SUr 41, adult female): Finland: Jokilampi, September 8, 1986, coll. M. Cieślak (Figure 1(g)).

Sixteen females, nine males, and specimens were preserved in alcohol from the quill of small inner secondaries (host no. SUr 47, adult male); Sweden: Järsjö, July 2, 2007, coll. M. Cieślak.

Four females and specimens were preserved in alcohol from the quill of UndrTC (host no. SUr 48, adult male); Poland: Cisna, October 11, 2004, coll. M. Cieślak (Figure 1(f)).

Two females and specimens were preserved in alcohol from the quill of small inner secondaries (host no. SUr 52, adult male); Sweden: Stavarn, July 4, 2008, coll. M. Cieślak (Figure 1(e)).

Four females, one male, and specimens were preserved in alcohol from quills of SGUppCs (host no. SUr 66, adult male); Finland: Oulu September 26, 2012, coll. M. Cieślak.

Three females and specimens were preserved in alcohol from quills of the scapulars coverts (host no. SUr 73, juvenile female); Finland: Ruka, August 21, 2014, coll. M. Cieślak (Figure 1(h)).

### 3.5. Prevalence and Feather Infestation of the Great Grey Owl

A total of 55 (26 males, 29 females) specimens were examined, including birds originating from Sweden and Finland. In total, 9900 feathers were examined (Table 1). A total of two host specimens were infested by quill mites, IP = 3.6%, CI = 0.6–12.4 (Table 2).

In the material consisting of 21 juveniles (in the 1st CY) and 34 adults (in the 2nd CY or older) birds, only two adult hosts were infested by quill mites (IP = 2.7%), an adult female from Sweden and an adult female from Finland.

Quill mites were found in 13 individual feathers. Each feather from a single host specimen had mites in five SGUppC nos. 1, 3, 4, 5 (host no. SN 24) and no. 2 (host no. SN 35), eight SGUndrC nos. 2, 3 4, 5 (host no. SN 24) and nos. 2, 3, 5, 6 (host no. SN 35) (Figures 1(i) and 1(j)).

### 3.6. Mite Material Examined Collected From the Great Grey Owl

Twenty-three females and three males mounted on slides and specimens preserved in alcohol from quills of secondary greater under and upperwing coverts (host no. AMU-DABE SNr 35, adult female); FINLAND: Ranua, 13 August 2005, coll. M. Cieślak.

Twenty-four females and three males mounted on slides and specimens preserved in alcohol from quills of secondary greater under and upperwing coverts (host no. AMU-DABE SN 24, adult female); SWEDEN: Vittjärv, 23 July 2004, coll. M. Cieślak (Figures 1(i) and 1(j)).

## 4. Discussion

*Bubophilus aluconis* was originally described from the tawny owl in England [14]. Later, it was also recorded on the African Wood Owl *Strix woodfordii* in Tanzania [17]. In other studies, the species was unexpectedly recorded from the long-eared owl, which is a type host for other *Bubophilus* species, that is, *B. asiobius* [15, 18]. The records of two new host species of the genus *Strix* presented in this paper, that is, the Ural owl and the great gray owl, show that this quill mite species belongs to the oligoxenous species infesting closely related host species (belonging to the same genus *Strix*). The record of this species on the long-eared owl is an example of host switching; the long-eared owl and tawny owl could potentially share the same habitat, such as nesting simultaneously within farm building complexes or establishing territories during the breeding season [18, 30].

Data about the proportion of a host population infested by quill mites (prevalence) are rarely reported in the literature. So far, it has been calculated based on the feathers collected from living or recently dead birds [31–38], kept on farms [39, 40], or in zoological gardens [41]. Some estimates of prevalences have been made based on specimens deposited in museums of natural history or scientific institutions [9, 19, 42–47]. In all of the abovementioned studies, quill mites were collected through an examination of either all flight feathers or only random feathers. The prevalence (IP) calculated through an examination of the whole plumage of the type habitat (all types of feathers potentially inhabited by the specific quill mite species (see habitat specificity in Skoracki [3, 4]) was recorded only for a few host species: (i) the Gregarious House Sparrow *Passer domesticus* infested with *Syringophiloidus minor* (Berlese), where the IP = 82% (*N* = 492) [31]; (ii) the Domestic Chicken *Gallus gallus domesticus* kept in crowded conditions and infested with *Syringophilus bipectinatus* Haller (IP = 75%, *N* = 1,500) [40]; (iii) the Ovenbird *Seiurus aurocapilla* infested with *Betasyringophiloidus seiuri* (Clark) (IP = 42.9%, *N* = 21) [34]; (iv) the Rock Ptarmigans *Lagopus muta* infested with *Mironovia lagopus* Bochkov and Skirnisson (IP = 7.3%, *N* = 1.209) [38]; and (v) the Boreal Owl *Aegolius funereus* infested with *Bubophilus aegolius* (IP = 7.3, *N* = 55) [19]. Obviously, only an examination of all hosts' feathers gives a real score of the IP, whereas random sampling can provide approximate or lowered values [34, 46]. As we can see, the highest prevalence was noted among social and domestic birds, where a horizontal transfer may play an important role in the migration of quill mites. On the other hand, our results show a relatively low prevalence for the tawny owl, the Ural owl, and the great grey owl, where mites *Bubophilus aluconis* use only a small fraction of individual hosts available in the environment. This phenomenon may result from the hosts' antisocial behaviour.

Truth be told, we expected a high quill mite prevalence on the tawny owl, because it is the most common, widespread, and abundant owl species in the Western Palearctic [20–23, 48, 49]. Thus, one might assume that contacts between individuals would also be high and would favour new mite infections. Paradoxically, this is not the case: as a resident species, a pair of tawny owls are strongly territorial, fiercely defending their territory (including food resources), and the male and female reside there almost all year round, deliberately avoiding contact with other individuals, which they treat as a threat [20–23, 48, 49].

The second host species, the Ural owl, is a generalist feeder, but its reproduction is highly dependent on 3–4-year cyclic fluctuations of microtones [20–23]. Like the tawny owl, it is a solitary and territorial resident species whose survival is linked to the possession of an exclusive year-round feeding territory acquired by its juveniles in autumn. It is a moderately numerous species [48, 49]. On the other hand, food availability is the main factor determining the reproduction of the great grey owl, which can breed once every 3 or 4 years. However, they may not reproduce when food is scarce [20–23, 48, 49].

Still, the low production of new individuals and the small number of contacts of adult birds during and outside the breeding season are not conducive to the development of new mite infestations. Therefore, we speculate that in the system composed of the mite *Bubophilus aluconis*, the tawny owl, and the great grey owl, a horizontal transfer (if present) only plays a marginal role. The situation may be different in the case of the Ural owl. Currently, we observe that southern populations in Central Europe and parts of Scandinavia are characterised by high dynamism, manifested by an increase in the number of breeding pairs of this species [50–54]. Compared to previous assessments, we observe a real increase, which does not only result from a better diagnosis of the species occurrence [50–54]. As a consequence, local populations, pairs, and individuals of the tawny owl are chased away from their territories or killed (the tawny owl becomes prey of the predatory Ural owl) [20, 49]. In this way, the Ural owls take over breeding territories, hollows, and foraging areas of the tawny owl [20, 49].

So, while killing tawny owls, they have direct contact with individuals infested by mites. Therefore, we assume this can be an additional way for infestations of the Ural owl. This type of quill mite transmission between the predator and the prey was documented by Nettress [7]. Apart from a large number of *Bubophilus aluconis* in the tawny owl's feathers, Nattress [7] also found two specimens of *Syringophilopsis kirgizorum*, which is a specific parasite for finches (Fringillidae) and points out that the diet of the Tawny Owl in woodlands comprises mainly small rodents, birds, amphibians, shrews, earthworms, and beetles, whereas in towns, the diet is mainly composed of birds, small rodents, and other available prey [7].

Another surprising route of transmission may be mixed broods between the tawny owl and the Ural owl (recently discovered, tawny owl owlet as an uninvited guest in the Ural owl brood) [55]. Additionally, the mites can survive in nest hole litter material left behind by the tawny owl, which the Ural owl reuses for breeding, making it also a likely way of infestation. Such an example was presented by Kivganov and Sharafat [56], who described the quill mite species *Columbiphilus khushalkhani* found in the nest of the common pigeon *Columba livia*. Interestingly, this quill mite species infests mainly representatives of galliform birds, whereas the common pigeon is regarded as an accidental host [4].

What is worth noticing is the fact that in the host sample of the tawny owl, only one juvenile bird (in 1st CY) and one adult bird (2nd CY or older) were parasitised with quill mites. In the case of the Ural owl, two juveniles and seven adults were infested. In contrast, no infestation with parasites was found in juvenile birds of the great grey owl, but only two adults. This situation may be due to the high mortality of quill mites during the moulting period, which lasts from early May to late June for the tawny owl, late April to late May for the Ural owl, and early June to late September for the great grey owl [23, 48, 49, 57].

Typically, during the moulting passage, adult mite females emerge from mature feather quills (adult birds), disperse, and enter new feathers (newly grown) of nestlings. [2, 5, 6, 31, 32]. Between these periods, the growth and dispersal of the population of quill mites must be synchronised with critical events in the host's life cycle in order to minimise the loss of dispersants [6, 31, 32]. The absence of infested juvenile birds of the great grey owl indicates that replacements of feathers have a significantly negative effect on the number of mite dispersants which successfully enter the newly developed feathers [24, 31, 57, 58]. Therefore, there is no perfect moulting time for mite dispersion, and when one feather is removed, a new one is not always immediately available [6, 24, 31, 32, 57, 58].

Most parasites shorten, as much as possible, their stay in the external environment during the transmission phase [31, 32, 58]. However, recent observations have shown that quill mites are able to wait a certain amount of time on the host's body until a potential new feather appears (Skoracki, unpublished data). Moreover, when the chicks are in the nest, any feather growing on their body may be potentially infested by mites [6, 19, 24]. Therefore, the lack of infested juvenile great grey owls in our sample does not prove that there is no such transfer between adult birds and young birds. It may only mean that they have not been identified and described [19, 24, 31, 32, 57].

### 4.1. Habitat Preference

Syringophilids live in quills of various parts of plumage (P, S, alulars, coverts, tail-feathers, and contour feathers), and particular species' preference differs in the kind of inhabited plumage (niche or habitat specificity). This division in habitat preference is already visible on the subfamily level, where representatives of the subfamily Picobiinae exclusively inhabit contour feathers, whereas mites of the subfamily Syringophilinae occupy mainly quills of flight feathers. Among Syringophilinae, some species prefer only S, only P, or only coverts, even on the same host specimen [2, 3]. The type of niche where particular mite taxa can live is determined by two feather characteristics, such as the volume of the quill and its wall thickness [2, 34, 59]. Syringophilinae females with short chelicerae inhabiting large quills are not able to pierce the quill wall and inevitably die. On the other hand, when quills are too small, mites cannot produce an adequate number of propagules to disperse to a new host. Due to the fact that there is a correlation between the volume of the quill, the thickness of its wall, the size of the mite, and the length of its chelicerae, an optimal niche is the one that can house a relatively large number of mites (even over 100 individuals per quill), and its wall thickness does not interfere with foraging [2, 59].

Therefore, the issue of how dispersant quill mites are able to find and enter feathers that provide the most advantageous range is rather mysterious, although some sensory elements situated at the tip of palps (chemo-mechanoreceptor palpal sensilla) of these mites can play a fundamental role in the search for the umbilicus superior in the developing feather and in the detection of a place for piercing the quill wall [5, 60]. As shown above, quill mites use only a small fraction of the host population available in the environment, a phenomenon which can be explained by the solitary behaviour of owls. Similarly, our results indicate that *Bubophilus aluconis* uses very limited types of habitats in the plumage of the hosts. In our study, we found syringophilid mites only in the inner S of the tawny owl, secondary greater under and upperwing coverts of the great grey owl, and inner Ss, primary and secondary upperwing coverts, UndrTC, and coverts from the scapulars of the Ural owl. Nevertheless, the question of why the *Bubophilus aluconis* selects these feathers is more of a challenge. We suppose that the absence of these parasites in the studied P, S, and R indicates that these types of feathers are unsuitable for these mites because the feathers' walls are too thick to be pierced and to feed successfully. Consequently, *Bubophilus aluconis* occupy only smaller feathers, like wing and tail coverts or only smaller flight feathers, that is, the most inner S.

Currently, it is believed that the main way of spreading mites in feathers is the period of their replacement by the bird, that is, moulting [2, 6]. The moult sequence of *Strix* owls in the wild is similar; however, not all flight feathers are moulted every year, but usually every 2 years (less often three). On the other hand, contour feathers covering the entire body are replaced completely every year. First, in some P, the upper and underwing coverts are replaced, then the upper parts of the wings and parts of the chest. Moulting of P starts with the inner ones, most often from P2, sometimes from P1, yet three inner P may be shed simultaneously or it starts from P5 or P6. S are replaced in four series: S12-S8, S13-S16, S1-S4, and S5-S7, but only inner Ss S13-S16 are replaced every year. R are shed irregularly at later data within a short period. Finally, feathers on the head and belly are replaced [24, 48, 49, 57, 61, 62]. For instance, inner S (S13-S16), primary and secondary upper and underwing coverts, UndrTC, and the scapulars of nestlings and adult birds stop growing first [24, 57]. Perhaps this observation could help explain why the observed prevalence of *Bubophilus aluconis* in juvenile and adult owls was high in these feathers. Basically, during the “nestling passage,” *B. aluconis* is likely to select inner S, wing coverts, and scapular feathers of the nestling due to a combination of feather size suitability (relatively small wing feathers) and the expected juvenile feather lifespan (at least 1 year). Most importantly, however, these feathers grow the fastest so it can be assumed that the direct reason for the infestation of these feathers is their rapid growth on a young individual, thus creating optimal conditions for parasites [19, 24, 57]. On the other hand, we cannot exclude the notion that all P and secondaries from S1 to S10 represent an inadequate habitat for quill mites, because the quill wall is too thick, and they are not able to feed. Moreover, we cannot exclude the migration of quill mites from adults' flight to contour feathers that cover the whole body; however, this aspect has not been analysed in the present study.

Although the relatively low prevalence of the *Bubophilus aluconis* can result from an array of simultaneous factors, for example, the biology of the host's moult, feather structure, specific breeding, and the behaviour of the tawny owl, Ural owl, and great grey owl, an alternative explanation cannot be disregarded as the system composed of the mite *B. aluconis*, and the host *Strix* genus represents an evolutionarily young and not very stable system. In this scenario, *B. aluconis*, as a straggler that reaches a new host, has a limited ability to survive, reproduce, and disperse [63, 64].

## 5. Conclusion

The issue requires further studies on the quill mite fauna of 22 other representatives of the genus *Strix* [65, 66]. Furthermore, we acknowledge the necessity of experimental studies to provide direct evidence supporting our hypotheses regarding the mechanisms of distribution, dispersion, and preference of mites colonising feathers. Only through such empirical results can we gain confidence that our assumptions align with the observations in the feathers of the hosts under investigation. However, conducting such research in controlled ecological conditions is a challenging task due to the scale and size of the mites involved. Currently, we lack established and refined protocols for conducting such studies under controlled settings.

## Figures and Tables

**Table 1:** Number and type of feathers examined.

**Type of the feathers (** **N** **)**	**Strix aluco (** **N** = 77**)**	**Strix uralensis (** **N** = 75**)**	**Strix nebulosa (** **N** = 55**)**
Primaries *N* = 10	1540	1500	1100
Secondaries *N* = 16	2464	2400	1760
Rectrices *N* = 12	924	900	660
Primary greater upperwing covert (PGUppC), *N* = 10	1540	1500	1100
Primary greater underwing covert (PGUndrC), *N* = 10	1540	1500	1100
Secondary greater upperwing covert (SGUppC), *N* = 14	2156	2100	1540
Secondary greater underwing covert (SGUndrC), *N* = 14	2156	2100	1540
Uppertilte covert (UppTC) *N* = 10	770	750	550
Undertilte covert (UndrTC) *N* = 10	770	750	550

**Table 2:** Host species of the genus *Strix* infested with quill mite *Bubophilus aluconis* with the prevalence, types of infested feathers, and distribution.

**Host species**	**No of examined/infested**	**Prevalence and confidence interval**	**Type of infested quill feathers**	**Locality**
*Strix aluco*	77/2	2.6%; 0.5–8.9	Inner secondaries (S14)	Poland and Sweden
*Strix uralensis*	75/9	12%; 6.2–21.2	Inner secondaries, primary greater upperwing coverts (PGUppC), secondary greater upperwing coverts (SGUppC), undertail coverts (UndrTC), covers from the scapulars	Poland, Sweden, and Finland
*Strix nebulosa*	55/2	3.6%; 0.6–12.4	Secondary greater under and upperwing coverts (SGUppC, SGUndrC)	Finland and Sweden

## Data Availability

The collected mite specimens data used to support the findings of this study are available from the corresponding author upon request. Data sets utilized for this research were retrieved from AMU-DABE. This feather material is currently deposited in the Department of Avian Biology and Ecology, Faculty of Biology, Adam Mickiewicz University, Poznań, Poland (UAM). All collected mite specimens are deposited in the Adam Mickiewicz University, Department of Animal Morphology, Poznan, Poland (UAM).
